# Differential Effects of STCH and Stress-Inducible Hsp70 on the Stability and Maturation of NKCC2

**DOI:** 10.3390/ijms22042207

**Published:** 2021-02-23

**Authors:** Dalal Bakhos-Douaihy, Elie Seaayfan, Sylvie Demaretz, Martin Komhoff, Kamel Laghmani

**Affiliations:** 1 Centre de Recherche des Cordeliers, INSERM, Sorbonne Université, USPC, Université Paris Descartes, Université Paris Diderot, 75006 Paris, France; dalal.bakhos_aldouaihy@sorbonne-universite.fr (D.B.-D.); elie.seaayfan@uni-marburg.de (E.S.); sylvie.demaretz@sorbonne-universite.fr (S.D.); 2 CNRS, ERL8228, 75006 Paris, France; 3 University Children’s Hospital, Philipps-University, 35043 Marburg, Germany; Martin.Koemhoff@uk-gm.de

**Keywords:** NKCC2, ERAD, STCH, Hsp70, membrane, trafficking, Bartter syndrome, hypertension

## Abstract

Mutations in the Na-K-2Cl co-transporter NKCC2 lead to type I Bartter syndrome, a life-threatening kidney disease. We previously showed that export from the ER constitutes the limiting step in NKCC2 maturation and cell surface expression. Yet, the molecular mechanisms involved in this process remain obscure. Here, we report the identification of chaperone stress 70 protein (STCH) and the stress-inducible heat shock protein 70 (Hsp70), as two novel binding partners of the ER-resident form of NKCC2. STCH knock-down increased total NKCC2 expression whereas Hsp70 knock-down or its inhibition by YM-01 had the opposite effect. Accordingly, overexpressing of STCH and Hsp70 exerted opposite actions on total protein abundance of NKCC2 and its folding mutants. Cycloheximide chase assay showed that in cells over-expressing STCH, NKCC2 stability and maturation are heavily impaired. In contrast to STCH, Hsp70 co-expression increased NKCC2 maturation. Interestingly, treatment by protein degradation inhibitors revealed that in addition to the proteasome, the ER associated degradation (ERAD) of NKCC2 mediated by STCH, involves also the ER-to-lysosome-associated degradation pathway. In summary, our data are consistent with STCH and Hsp70 having differential and antagonistic effects with regard to NKCC2 biogenesis. These findings may have an impact on our understanding and potential treatment of diseases related to aberrant NKCC2 trafficking and expression.

## 1. Introduction

The electroneutral solute carrier family 12A (SLC12A) transport family encompasses several branches of homologous genes [1]; each of them encodes unique cation-chloride co-transporter (CCC). The kidney Na-K-2Cl co-transporter (NKCC2), which is encoded by the SLC12A1 gene [2,3], is expressed in the apical membrane of ascending limb of Henle epithelial cells (TAL) and the macula densa [4]. NKCC2 is the pacemaker of TAL sodium chloride reabsorption which accounts for 20–30% of the filtered load of NaCl [1]. It plays therefore a pivotal role in urinary concentration and regulation of the blood pressure [1]. Given its high reuptake capacity, changes in NKCC2 transport activity can significantly alter renal NaCl reabsorption, leading eventually to disruption of the homoeostasis of salt and water handling [5,6]. Such changes in NKCC2 activity may be associated to the co-transporter itself (i.e., gain or loss-of-function mutations) or to alterations in the regulatory pathways that control NKCC2 trafficking and activity [1,7,8,9]. Indeed, loss of function mutations in *SLC12A1* can cause Bartter I syndrome, a rare genetic kidney disease marked by severe natriuresis with normal or low blood pressure, hypercalciuria, hyperparathyroidism, hypokalemia, and metabolic alkalosis [8,10,11]. Opposite to genetic inactivation of NKCC2, enhanced NKCC2 activity has been associated with salt-sensitive hypertension in animal models and in humans, in particular African-Americans [1,12,13,14]. NKCC2 is the target of loop diuretics such as furosemide and bumetanide, commonly used to lower BP [15]. Moreover, a large genetic screening in participants of the Framingham Heart Study revealed that rare and subtle heterozygous mutations of the *SLC12A1* gene, protect against hypertension, further proving that factors regulating the activity of NKCC2 are key determinants of the control of BP in the general population [16,17]. All of these data emphasize the important role of NKCC2 in electrolytes homeostasis and blood pressure control in normal physiology and in pathogenesis of Bartter syndrome. Despite the major role of NKCC2 in the regulation of sodium balance and blood pressure, the underlying mechanisms and respective protein networks regulating its transport activity at the molecular level namely in its trafficking, targeting, and turnover remain largely unidentified.

In eukaryotes, about 40% of the cell’s proteins are either membrane bound or secretory and are therefore directed to the endoplasmic reticulum (ER), where they endure a dynamic folding environment and withstand the rigorous monitoring of quality-control pathways to reach functional maturity [18,19]. Only correctly folded proteins are transferred to the Golgi apparatus for maturation [19,20]. Consequently, mature proteins are targeted to their final destination, they are directed to the plasma membrane or in organelles of the secretory and endocytic compartments, or they are secreted from the cell. When the folding process fails, defective proteins are dislocated across the membrane for cytosolic proteasome degradation through mechanisms known as ERAD (endoplasmic reticulum-associated degradation) [19,20]. Both wild type and mutant variants of membrane proteins with complex topologies are prone to ER quality control and degradation. The chloride channel protein CFTR (cystic fibrosis transmembrane conductance regulator), characterized as the first integral membrane mammalian ERAD substrate, is the most extensively studied example [21,22]. Given its complex and inefficient folding pathway, the vast majority of newly synthesized wild type CFTR protein is selected for ERAD [21,22], as is the epithelial sodium channel, ENaC [23] and the thiazide-sensitive NaCl co-transporter (NCC) [24]. The low folding efficiency is further decreased by mutations as seen in the most frequent mutation of CFTR, (CFTRΔF508), which is sufficient to retain almost all of the protein in the ER and targeted for ERAD, leading to cystic fibrosis [21,22]. ERAD is a multistep mechanism comprising the recognition, targeting, retranslocation, extraction, ubiquitination, and degradation by the proteasome. In general, the selection of proteins for ERAD involves substrate-specific interactions with molecular chaperones such as shock protein 70 (Hsp70) and 40 (Hsp40) families, and with co-chaperones regulators [25,26]. Similar to ENaC and CFTR, our previous studies provided evidence for NKCC2 as a substrate for an ERAD quality control system [27,28,29,30]. We have previously showed that similar to CFTR, the vast majority of newly synthetized NKCC2 protein is retained in the ER and destined for ERAD very likely because the protein is unable to fold correctly limiting therefore its export from the ER and its traffic to the cell membrane [28,29,30]. In that pathway, the protein lectin OS9, which interacts with the C-terminal tail of NKCC2, can function to promote the degradation of the co-transporter by the proteasome [28]. Moreover, melanoma-associated antigen D2 (MAGE-D2), another regulator of NKCC2 biogenesis, recently identified mutated in patients with transient Bartter syndrome by our team, has been also implicated in the ER quality control of NKCC2 [27]. Indeed, we previously showed that MAGE-D2 mutations lead to a very severe form of antenatal Bartter syndrome by altering the expression of NKCC2 and its structurally related kidney Na-Cl co-transporter NCC [27]. With this regard, we suggested that MAGE-D2 is very likely to cooperate with Hsp40, a binding partner of NKCC2 and a key modulator of protein folding and stability to regulate the ERAD of the co-transporter [27]. Although all these data clearly highlight the fact the ER quality control is a critical step in NKCC2 trafficking to the cell surface, very little is known about the specific molecular determinants involved in this process. In this study, we sought to identify additional factors and partners that target NKCC2 for ER-associated degradation and could help to define more the mechanisms by which NKCC2 is subject to ERAD. Here, we identified STCH and the stress inducible Hsp70 (Hsp72/HSPA1A) as two novel binding partners of NKCC2. The stress-inducible Hsp70 (Hsp72/HSPA1A) is commonly simply named Hsp70. STCH is also known as “microsomal stress 70 protein ATPase core” and “heat shock 70-kDa protein member 13” (HSPA13) [31]. STCH is constitutively expressed in all human cell types and is induced by incubation with calcium, but not by exposure to heat shock [31,32]. In contrast to the extensively studied stress-inducible Hsp70 [33,34], only few studies have linked STCH to some diseases [35,36,37,38] and its major cellular and molecular function has remained poorly defined. Here, we demonstrate that STCH and Hsp70 interact with the immature form of NKCC2 mainly at the ER. Importantly, we provide evidence that STCH and Hsp70 differentially regulate the expression of NKCC2 and its disease-causing mutants. Interestingly, we show that STCH promotes efficient ER associated degradation of NKCC2 in a proteasome and lysosome dependent manner, revealing therefore a new molecular pathway in the regulation of the co-transporter. Taken together, these findings may open new avenues in studying the regulation of NKCC2 quality control and in ultimately identifying new “druggable” targets to prevent and/or treat kidney disorders related to sodium balance in general, and type I Bartter syndrome in particular.

## 2. Results

### 2.1. Identification of STCH as a Novel NKCC2 Binding Partner

**Figure 1:**
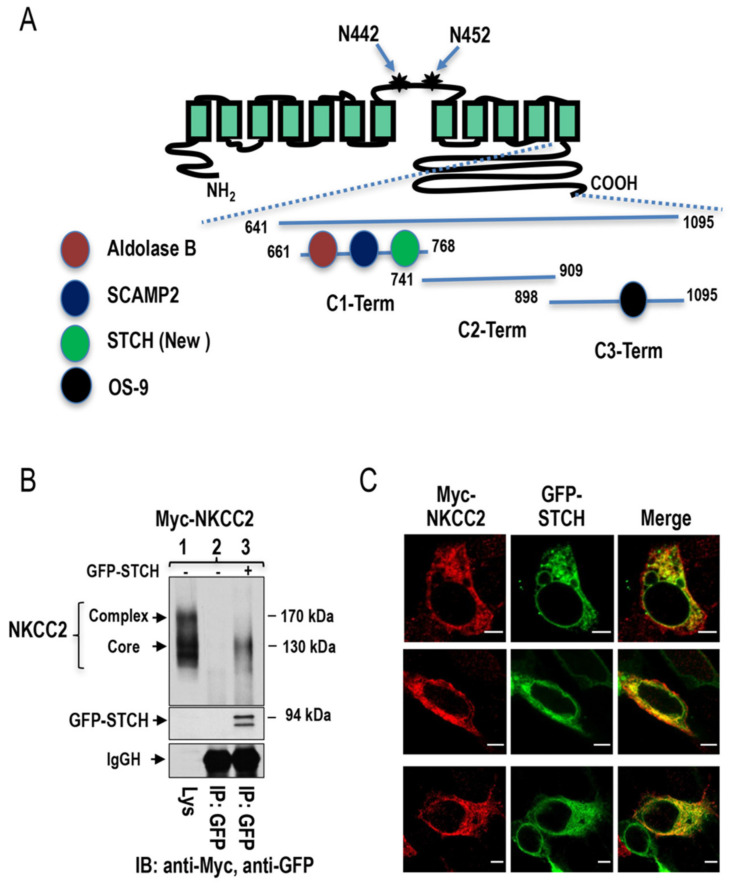
Identification of STCH as a novel NKCC2-interacting protein. (**A**) Mouse NKCC2 yeast two-hybrid baits constructs. A proposed topology for sodium-coupled chloride co-transporter NKCC2. N442 and N452 are the potential *N*-glycosylation sites. As previously described, mouse NKCC2 C terminus was divided into three peptide fragments (C1-term, C2-term, and C3-term) used as baits for the yeast two-hybrid. Similar to Aldolase B [39] and SCAMP2 [40], STCH interacts with C1-term while OS9 binds to C3-term [28]. (**B**) NKCC2 binds, in vivo, to STCH in HEK cells. HEK cells transiently transfected with Myc-NKCC2 singly or in combination with GFP-STCH were immunoprecipitated (IP) with anti-GFP anti-body (**lanes 2 and 3**). 5% of total cell lysate (Lys) was resolved as positive control. Co-immunoprecipitated NKCC2 and STCH proteins were detected by immunoblotting (IB) using anti-Myc (**lane 3**) and anti-GFP respectively (**lane 3**). IgGH, the heavy chain of IgG. The positions of immature (core glycosylated) and mature (complex-glycosylated) proteins of NKCC2 are indicated. The interaction of NKCC2 with STCH involves mainly the immature form of the co-transporter. (**C**) Imunofluorescence confocal microscopy showing distribution of Myc-NKCC2 and GFP-STCH in HEK cells. Fixed and permeabilized cells were stained with mouse anti-Myc for NKCC2 (Texas Red). The yellow color (merged image) indicates co-localization of the proteins. Bars, 5 μm.

To identify novel NKCC2-binding partners, we undertook a yeast two-hybrid (Y2H) screening of a human kidney cDNA expression library, using a sequence of bait fragments comprising the predicted cytoplasmic C terminus (residues 661–1095) of murine NKCC2 termed C1-term (residues 661–768), C2-term (residues 741–909), and C3-term (residues 898–1095) as shown in Figure 1A and mentioned in previous studies [28,39,40]. Several positive clones encoding putative interacting NKCC2 proteins were identified from the screen. Among these candidates, one clone (number # 62) encoding Hsp70-like stress 70 protein chaperone (STCH) was isolated as a novel and specific binding protein of the proximal region (C1-term) of NKCC2 terminus (Figure 1A). To determine whether STCH interacts with NKCC2 in mammalian cells, we performed co-immunoprecipitation studies. To this end, HEK cells were transiently transfected with Myc-NKCC2 plasmid expressed either singly or in combination with GFP-STCH construct and cell lysates were incubated with anti-Myc or anti-GFP antibody. The resultant immunoprecipitates were resolved by SDS-PAGE and immunoblotting. As we previously described [39,40], exogenously expressed NKCC2 in renal cultured cells is detected as two major bands, one of ~130 kDa and another of ~170 kDa, corresponding to the core/high-mannose and complex N-glycosylated forms of the co-transporter, respectively (Figure 1B). In coimmunoprecipitation experiments, NKCC2 was detected in the precipitation complex containing anti-GFP antibody and obtained from cells co-transfected with NKCC2 and GFP-STCH (Figure 1B, *lane 3*), but not from cells transfected only with NKCC2 alone (Figure 1B, *lane 2*), indicating that STCH specifically interacts with NKCC2 protein. Interestingly, as can be seen in Figure 1B (*lane 3*), this interaction between STCH and NKCC2 involves mainly the immature and ER-resident form of NKCC2. Altogether, these results not only confirm the interaction between NKCC2 and STCH revealed by Y2H but also clearly show that STCH interacts mainly with the core glycosylated and immature form of NKCC2 in vivo.

To further confirm these findings, we performed co-localization studies. To this end, immunofluorescence was performed in HEK cells co-expressing Myc-NKCC2 and GFP-STCH proteins. Of note, we have previously demonstrated that N-terminal tagging of NKCC2 with myc does not affect the co-transporter trafficking [39,40]. As shown in Figure 1C, myc-NKCC2 (red) co-localized with STCH (green) and their immunofluorescence staining pattern seems to be limited to a perinuclear ER-like distribution, which is consistent with STCH binding mainly to the immature and ER resident form of NKCC2. In sum, these findings are in accordance with the results of Y2H and co-immunoprecipitation studies and strongly suggest that NKCC2 and STCH interact mainly at the ER.

### 2.2. Site of NKCC2 Interaction with STCH

**Figure 2:**
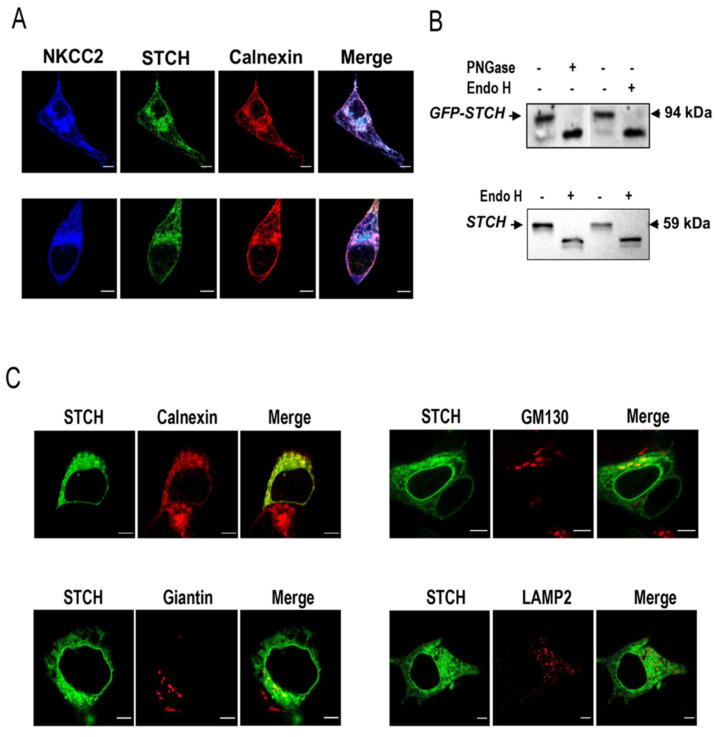
STCH co-localizes with NKCC2 mainly at the Endoplasmic Reticulum. (**A**) Intracellular localization of NKCC2 and STCH in HEK cells. All panels are fluorescence micrographs of HEK cells overexpressing NKCC2 tagged with myc and STCH tagged with GFP. After transfection, cells were fixed and immunostained with mouse anti-Myc and rabbit anti-calnexin (ER marker) antibodies, and analyzed using a confocal laser scanning microscope. The merge color indicates overlap between the Myc tag of NKCC2 protein (Alexa Fluor 647, Blue), the GFP tag of STCH (green) and the ER marker (Alexa Fluor 555, red) and represents co-localization of the proteins. Bars, 5 μm. (**B**) *N*-glycosidases digestion of STCH in HEK cells. Exogenous STCH-GFP (**upper panel**) and endogenous STCH (**lower panel**) in lysates from cells overexpressing STCH-GFP were digested with Endo H and/or PNGase F and analyzed by Western blotting. (**C**) Comparison between the cellular localization of STCH and several organelle markers. Cells transfected with NKCC2 tagged with myc and STCH tagged with GFP were fixed after transfection, immunostained with anti-calnexin (ER marker) or -Giantin (Golgi marker) or -GM130 (Cis Golgi marker) or -LAMP2 (Lysosomal marker) antibodies and visualized with GFP tag of STCH (green) and Alexa Fluor 555 conjugated secondary antibodies for each organelle marker. Analysis was performed by confocal laser scanning microscopy. Bars, 5 μm.

Previous studies indicated that STCH is localized mainly in the ER [31,32,41] which is in line with its interaction with the immature form of NKCC2. To verify this hypothesis, we next compared the subcellular distribution of NKCC2 and STCH with the ER marker calnexin. As illustrated in Figure 2A, in HEK cells co-transfected with Myc-NKCC2 and GFP-STCH, both proteins largely colocalized with the ER marker calnexin, supporting therefore further the notion that STCH binds to the immature form of the co-transporter and that the main site of interaction between the proteins is the ER. However, the fact that STCH interacts only with the immature form of the co-transporter cannot exclude the possibility that this interaction may also take place outside the ER. Indeed, given that the N-glycan of glycoproteins at the entrance of the Golgi apparatus is still of high mannose type, it is conceivable that the interaction of the two proteins may also take place at the cis-Golgi network. In support of this notion, Sleat et al. reported that STCH protein possesses an N-linked glycosylation site containing Man-6-P which per se, clearly indicates that STCH is not only N-glycosylated but it is also processed at least in the cis-Golgi network [42]. Indeed, lysosomal proteins such as the hydrolases are synthesized in the endoplasmic reticulum and move to the *cis*-Golgi network, where they are covalently modified by the addition of the mannose-6-phosphate (M6P) group [42]. We sought therefore to further characterize the profile of protein expression and subcellular distribution of STCH.

To discover the glycosylation status of STCH, lysates from HEK cells overexpressing GFP-STCH were treated with Endo H and PNGase. With this regard, it is worth mentioning that Endo H digestion provides an additional level of specificity in the analysis of Man-6-phosphorylation sites. Indeed, previous studies reported that Man-6-phosphorylation prevents further carbohydrate processing to Endo H resistant structures, and thus the vast majority of Man-6-P-containing glycans are Endo H-sensitive [43,44]. In contrast, PNGase F cleaves all N-linked oligosaccharides including complex type sugars that do not contain Man-6-P. Consequently, we anticipated that the major band detected around 94 kDa, corresponding to STCH (60 kDa) tagged with GFP (30 kDa) represents the Man-6 phosphorylated form of STCH and therefore should be Endo H sensitive. Importantly, as shown in Figure 2B (**upper panel**)*,* the band at 94 kDa (STCH plus GFP) was indeed sensitive to both Endo H and PNGase which is in line with the presence of STCH in the ER and/or its expression as a Man-6 phosphorylated glycoprotein. To confirm these findings, we checked also the glycosylation profile of endogenously expressed STCH in HEK cells. As shown in Figure 2B (**lower panel**) the detected band at 60 kDa, corresponding to the expected size of STCH, was also sensitive to Endo H, which is, again, in agreement with the expression of STCH as an ER-resident protein and/or its processing in the cis-Golgi-network to become Man-6 phosphorylated.

To further analyze the subcellular localization of STCH, we performed more co-localization studies using Golgi markers GM130 and giantin and the lysosomal marker LAMP2. As can be seen in Figure 2C (**upper left panel**), STCH intensively colocalized again with the ER marker calnexin clearly indicating that the main site of STCH expression is the ER. Interestingly, some STCH proteins appear to co-localize also with the cis-Golgi marker GM130 (Figure 2C, **upper right panel**) and to a lesser degree with the cis/medial Golgi marker giantin (Figure 2C, **lower left panel**), opening therefore the possibility of an additional interaction between NKCC2 and STCH at the cis-Golgi network. Importantly, STCH staining did not colocalize with the lysosomal marker LAMP2 (Figure 2C, **lower right panel**), which clearly indicates that although some STCH exits the ER apparatus and then become Man-6-phosphorylated in the transitional elements/cis-Golgi apparatus, they are not delivered to the lysosomes. Collectively, these findings provide additional evidence that STCH interaction with NKCC2 takes place mainly at the ER. Moreover, they also suggest that this interaction may additionally occur at the cis-Golgi compartment.

### 2.3. STCH Decreases NKCC2 Stability and Maturation

**Figure 3:**
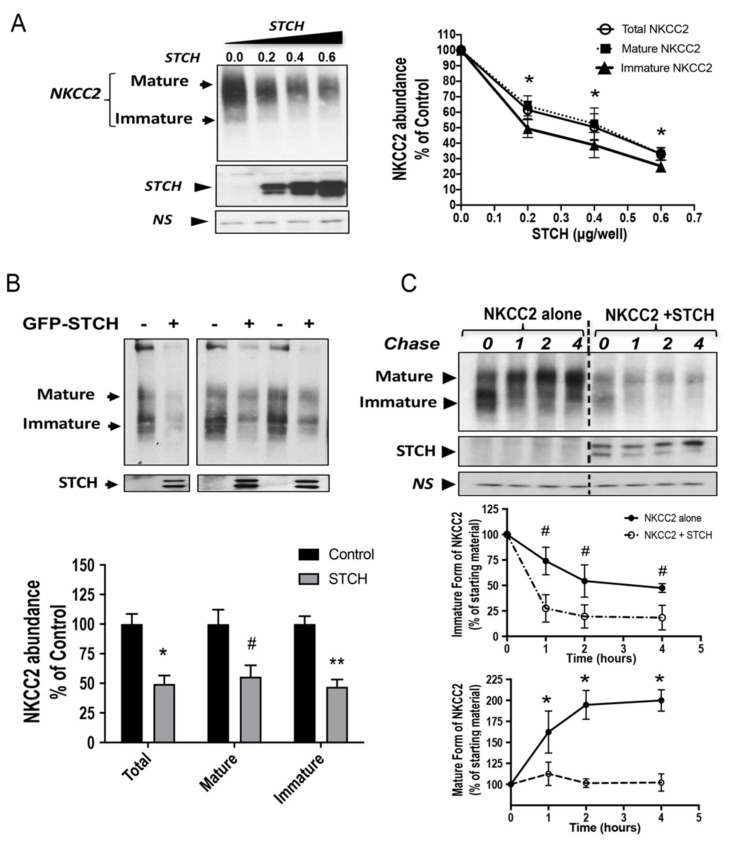
STCH alters NKCC2 stability and maturation. (**A**) Total NKCC2 protein abundance is reduced by STCH in a dose-dependent fashion. HEK cells were co-transfected with Myc-NKCC2 (0.2 μg/well) and increasing amounts of STCH (0.2–0.6 μg/well) as indicated. NKCC2 proteins were detected by Western blotting with Myc antibody (left panel). **Right panel**, densitometric analysis of total, immature, and mature NKCC2 proteins. Data are expressed as a percentage of control. *, *p* < 0.05 (*n* = 3). (**B**) STCH co-expression decreases the expression of NKCC2 proteins. **Upper panel**, representative immunoblot analysis showing the effect of STCH overexpression on NKCC2 protein abundance in HEK cells. Cells were transfected with Myc-NKCC2 alone (0.2 μg/well) or in the presence of GFP-STCH (0.6 μg/well). 16–18 h post-transfection, total cell lysates were subjected to immunoblot analysis for Myc-NKCC2 and anti-GFP. **Lower panel**, quantitation of steady state mature, immature, and total NKCC2 expression levels with or without STCH co-expression. Data are expressed as a percentage of control *±* SE, *, *p* < 0.04 (*n* = 4); #, *p* < 0.003 (*n* = 4); **, *p* < 0.002 (*n* = 4), versus control. (**C**) STCH decreases NKCC2 stability and maturation. **Upper panel**, representative immunoblot showing cycloheximide chase analysis of NKCC2 in the presence or absence of GFP-STCH. 14–16 h post-transfection, HEK cells transiently expressing WT NKCC2 alone or in combination with STCH, were chased for the indicated time after addition of cycloheximide. Total cell lysates were separated by SDS-PAGE and probed by anti-Myc antibodies. **Lower panels,** quantitative analysis of NKCC2 stability and maturation. The density of the mature and immature form of NKCC2 proteins was normalized to the density at time 0. #, *; *p* < 0.05 (*n* = 3) versus control. *NS*, a non-specific band illustrating the equal loading of protein extracts.

To determine the functional implications of STCH-NKCC2 interaction, we first tested the impact of STCH co-expression on total NKCC2 protein abundance. Accordingly, Myc-NKCC2 was transfected singly or with GFP-STCH in HEK293 cells. Then, 48 h post-transfection, total protein lysates were harvested and the protein levels of NKCC2 and STCH were analyzed by immunoblotting, using either anti-Myc or anti-GFP antibodies. As can be seen in Figure 3A, when increasing amounts of STCH (0.2–0.6 μg/well) were co-expressed with NKCC2 (0.2 μg/well), the protein levels of immature and mature forms of NKCC2 expression were reduced in a dose-dependent manner. To better visualize the expression of the immature form of NKCC2 during immunoblotting, we repeated these co-expression studies and performed cell lysis 16–18 h after cell transfection. As shown in Figure 3B, with 0.6 μg/well, STCH co-expression strikingly decreased the amount of immature and mature forms of NKCC2. As a consequence, total NKCC2 protein abundance was significantly decreased upon STCH co-expression (−51%; *p* < 0.001).

The interaction of STCH with the immature form of NKCC2 and the downregulation of the latter are likely due to changes in NKCC2 protein stability and maturation. To test this hypothesis, we analyzed the effect of STCH on the stability and maturation of NKCC2 using cycloheximide assays. Towards that end, 14 to 16 h after transfection of HEK cells with NKCC2 singly or in combination with STCH, cells were incubated with cycloheximide (CHX, 100 μM) for 0, 1, 2, and 4 h to block protein synthesis. Western blot assay was used to detect changes in NKCC2 protein maturation and degradation rate. During the chase period, under control conditions, the immature form of NKCC2 was progressively converted to a more slowly migrating band, representing the mature form. With this regard, it is worth emphasizing that we previously demonstrated that only the mature and complex glycosylated form of NKCC2 is able to reach the cell surface. As shown in Figure 3C (**middle panel**), STCH co-expression induced a rapid decay of the immature NKCC2 protein during the chase period. Of note, the decrease in half-life of immature NKCC2 form represents its degradation, as well as its conversion to the mature form of the co-transporter. Importantly, STCH co-expression heavily impaired the glycolytic maturation of NKCC2 by strikingly impeding protein conversion from immature to mature form (Figure 3C, **upper and lower panels**). Collectively, these data demonstrate that STCH promotes efficient NKCC2 degradation and that the immature form of the co-transporter is its preferred substrate, as evidenced by these cycloheximide chase data.

### 2.4. STCH Alters the Expression of NKCC2 Independently of the Expression System

**Figure 4:**
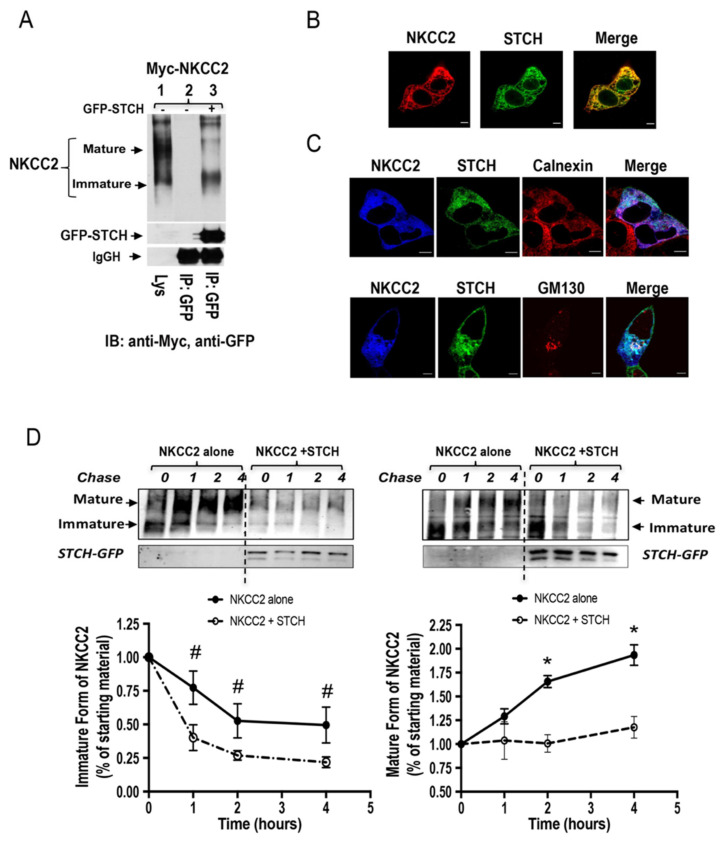
The effect of STCH on NKCC2 expression is independent of the expression system. (**A**) STCH interacts with immature NKCC2 in OKP cells. Cells were transiently transfected with Myc-NKCC2 either singly or in combination with GFP-STCH construct. Cell lysates were immunoprecipitated (IP) with anti-GFP antibody. NKCC2 protein was recovered from STCH immunoprecipitates mainly in its immature form (**lane 3**). (**B**) Imunofluorescence confocal microscopy showing distribution of Myc-NKCC2 and GFP-STCH in OKP cells. Cells were stained with mouse anti-Myc for NKCC2 (Texas Red). The yellow color (merged image) indicates co-localization of the proteins. Bars, 5 μm. (**C**) Similar to HEK cells, STCH and NKCC2 co-localizes mainly at the ER in OKP cells. All panels are fluorescence micrographs of OKP cells overexpressing myc-NKCC2 and GFP-STCH. **Upper panel**, fixed and permeabilized cells were stained with mouse anti-Myc and rabbit anti-calnexin (ER marker) antibodies. The merge color indicates overlap between the Myc tag of NKCC2 protein (Alexa Fluor 647, Blue), the GFP tag of STCH (green), and the ER marker (Alexa Fluor 555, red) and represents co-localization of the proteins. In **lower panel**, fixed and permeabilized cells were stained with mouse anti-Myc and rabbit anti-GM130 (cis-Golgi marker) antibodies. The merge color indicates overlap between the Myc tag of NKCC2 protein (Alexa Fluor 647, Blue), the GFP tag of STCH (green), and the cis-Golgi marker (Alexa Fluor 555, red) and represents co-localization of the proteins. In addition to the ER, the interaction between NKCC2 and STCH may also occur at the cis-Golgi. Analysis was performed by confocal laser scanning microscopy. Bars, 5 μm. (**D**) Analysis of NKCC2 stability and maturation monitored by cycloheximide-chase upon STCH expression. OKP cells were co-transfected with NKCC2 together with a control vector or GFP-STCH construct. Then, 14 h later, cell lysates were prepared at the indicated time points after cycloheximide treatment (100 μM). Total protein extracts are subjected to SDS-PAGE and probed using anti-Myc antibody. The density of the mature and immature forms of NKCC2 proteins was normalized to the density at time 0. #, *; *p* < 0.05 (*n* = 3) versus control.

Because the ERAD system and protein–protein interaction may be cell-dependent, we sought to corroborate our findings by conducting the experiments in a second renal cell line, OKP cells. As illustrated in Figure 4A, immunoprecipitation of STCH followed by immunoblotting for NKCC2 revealed again that the two proteins interact and that their association involves mainly the immature form of the co-transporter (Figure 4A, *lane 3*). Accordingly, STCH strongly colocalized with NKCC2 in OKP cells (Figure 4B). Moreover, similar to HEK cells, the main site of the interaction appears to be the ER as judged by the intensive colocalization of NKCC2 and STCH with the ER marker calnexin (Figure 4C, **upper panel**). Again, although NKCC2 and STCH co-localized mainly at the ER, some of the interaction between the two proteins seems to take place also at the cis-Golgi apparatus as judged by partial co-localization of NKCC2 and STCH with GM130 (Figure 4C, **lower panel**).

To assess the effect of STCH on NKCC2 biogenesis in OKP cells, we used again cycloheximide assay. As illustrated in Figure 4D, STCH overexpression decreased the stability of immature NKCC2 protein which is in agreement with the data described above in HEK cells. Again, the decrease in immature NKCC2 stability in cells overexpressing STCH was associated with a striking alteration in the conversion of the immature to the mature protein form. STCH-induced decrease in the maturation efficiency of NKCC2 was accentuated during the chase period to reach −81% at 4 h (*p* < 0.05) (Figure 4D, **lower right panel**). Taken together, these data demonstrate that, similar to HEK cells, STCH targets the immature form of NKCC2 to the ER associated degradation pathway in OKP cells, clearly indicating that STCH effects on the co-transporter expression are independent of the expression system. It is of great interest to note that, under identical experimental conditions, the overexpression of SCAMP2 or aldolase B, two others NKCC2 binding partner, had no effect on total NKCC2 protein abundance in OKP cells [39,40], strongly suggesting that the effect of STCH on total NKCC2 protein abundance is specific.

### 2.5. STCH Induces NKCC2 ERAD in a Proteasome-Dependent and Independent Manner

**Figure 5:**
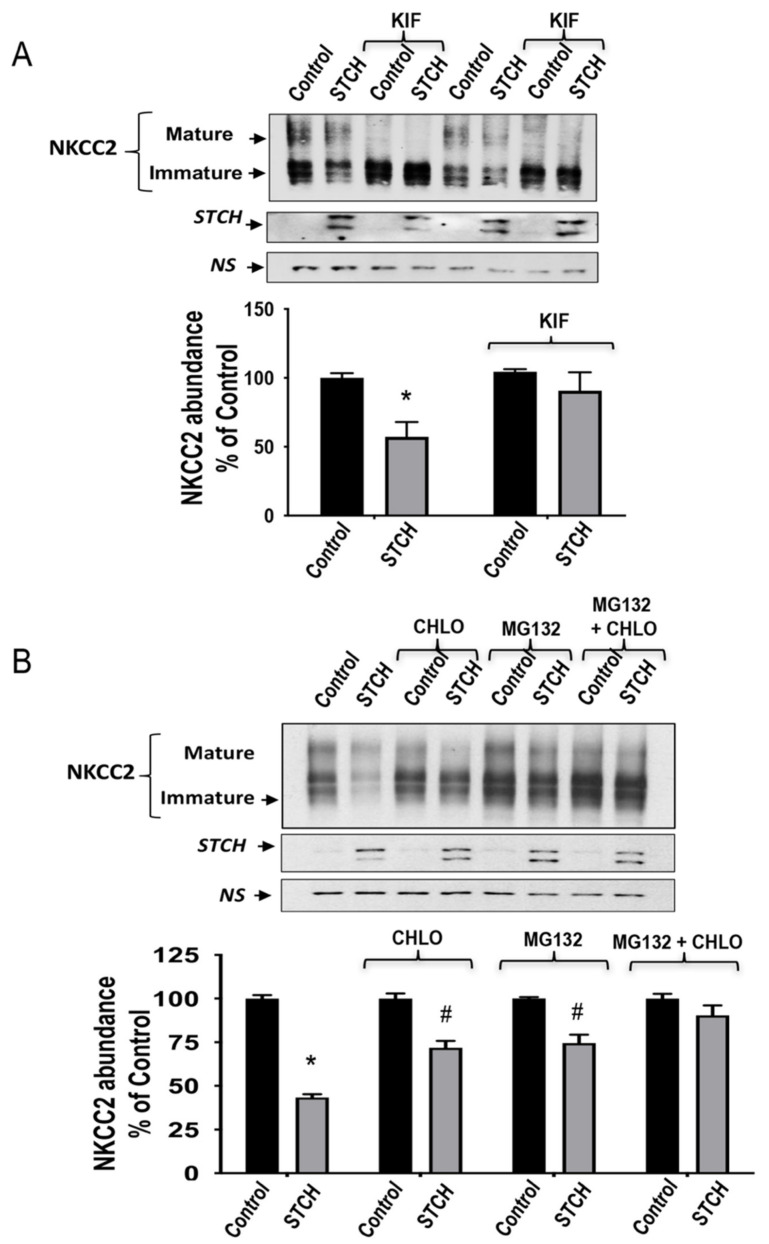
The ERAD of NKCC2 mediated by STCH involves both the proteasome and the lysosome. (**A**) Mannose trimming is required for STCH effect on NKCC2. OKP cells transiently transfected for with Myc-NKCC2 alone or with GFP-STCH, were treated with 25 μM of kifunensine (KIF) or without for 12–14 h prior to cell lysis. The cell lysates were subjected to SDS-PAGE and immunoblotted with anti-Myc and anti-GFP antibodies. **Bottom**, densitometric analysis of NKCC2 bands from untreated and treated cells with kifumensine (KIF). Data are expressed as percentage of control ± SE. *, *p* < 0.02 versus control (*n* = 3). (**B**) STCH decreases NKCC2 expression in a proteasome-dependent and lysosome dependent manner. 16 h post-transfection, HEK cells were treated with or without 2 μm MG132 or 100 μm chloroquine for 6 h prior to cell lysis. The cell lysates were subjected to immunoblotting with anti-Myc and anti-GFP antibodies. **Bottom**, densitometric analysis of NKCC2 bands from untreated and treated cells with MG132 or chloroquine (CHLO). Data are expressed as percentage of control ±S.E. #, *p* < 0.05 versus control (*n* = 3). *NS*, a non-specific band illustrating the equal loading of protein extracts.

Based upon the above findings, we hypothesized that STCH association with NKCC2 promotes endoplasmic reticulum-associated degradation of immature misfolded NKCC2, leading to a decrease in total expression and maturation of the co-transporter. Accordingly, investigations of ERAD inhibition and consequent probable changes in NKCC2 expression were conducted in cells treated with kifunensine, which inhibits α-mannosidases I (including ER mannosidase I and EDEM) required for the early steps of ERAD misfolded glycoprotein recognition. Towards this end, cells were transfected with Myc-NKCC2 cDNA in the absence (empty vector) or presence of STCH-GFP construct for 5 h before treatment with kifunensine overnight. Again, given that the data described above clearly demonstrate that the immature form of NKCC2 is the preferred substrate of STCH, cell lysis was performed 16–18 h post-treatment to better visualize the immature form of the co-transporter during immunoblotting analysis. As can be seen in the upper panel of Figure 5A, kifunensine (KIF) increased total expression of immature N-glycosylated NKCC2, as expected following impaired mannose trimming in the ER. Most importantly, upon treatment with kifunensine, the STCH-dependent decrease of NKCC2 protein level was abrogated (Figure 5A,B). Hence, these data clearly indicate that STCH-enhanced degradation of NKCC2 requires mannose trimming by α-mannosidase I to target misfolded NKCC2 for ERAD.

It is well known that ERAD involves, in most cases, activation of the proteasome proteolysis pathway and/or the lysosomal machinery. Therefore, to explore the possible implication of proteasomal and/or lysosomal degradation pathways in the STCH-induced down-regulation of NKCC2, 16 h post-transfection, cells were treated for 6 h with 2 μm MG132 or 100 μm chloroquine and their lysates were subjected to Western blotting analysis. In accordance with our previous work, MG132 treatment significantly increased the protein levels of the immature form of NKCC2 without a notable increase in its mature form, an effect consistent with an ER-associated degradation. Interestingly, in cells overexpressing STCH, MG132 appeared to increase NKCC2 levels and to exert a protective effect against STCH-induced NKCC2 degradation (Figure 5B), suggesting that a proteasome-dependent mechanism underlies the STCH mediated degradation of NKCC2. However, MG132 alone failed to completely suppress NKCC2 degradation elicited by STCH (Figure 5B, **lower panel**). Interestingly, in the presence of chloroquine, STCH effect on NKCC2 was also partially inhibited revealing therefore that the lysosomal machinery is also involved in STCH-induced down-regulation of the co-transporter. Accordingly, the combined use of MG132 and chloroquine was able to abrogate the STCH induced downregulation of NKCC2, and to fully restore the expression level of the co-transporter. Altogether, these findings strongly suggest that STCH decreases NKCC2 expression by promoting its ERAD in a proteasome and lysosome dependent manner.

### 2.6. Stress-Inducible Hsp70 Interacts Also with NKCC2

**Figure 6:**
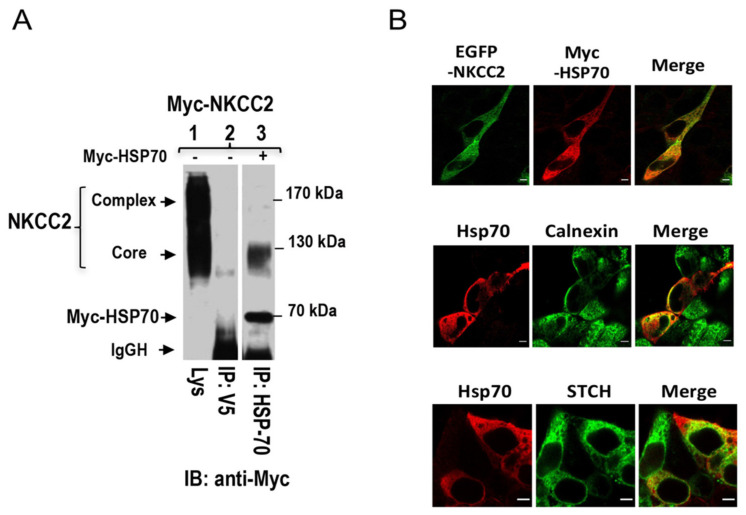
NKCC2 interacts with the stress-inducible Hsp70. (**A**) Hsp70 interacts also with immature NKCC2. Cell lysates from OKP cells transiently transfected with Myc-NKCC2 singly or in combination with of Myc-Hsp70 were immunoprecipitated (IP) with anti-Hsp70 or anti-V5 antibody. NKCC2 protein was recovered from Hsp70 immunoprecipitates only in its immature form (**lane 3**). (**B**) Similar to STCH, Hsp70, and NKCC2 co-localizes at the ER. **Upper panel**, immunofluorescence confocal microscopy showing distribution of NKCC2 and Hsp70 in HEK cells. Transiently transfected HEK cells with EGFP-NKCC2 and Myc-HSP70, were fixed, permeabilized, and then stained with mouse anti-Myc for NKCC2 (Texas Red). The yellow color (merged image) illustrates co-localization of the proteins. **Middle panel**, HEK cells transfected with Myc-Hsp70 were stained with mouse anti-Myc (Texas Red; red) and rabbit anti-calnexin (FITC; green). Yellow indicates overlap between Hsp70 (red) and the ER marker (green). **Lower panel**, STCH colocalizes with Hsp70. HEK cells transiently transfected with GFP-STCH and Myc-Hsp70, were fixed and permeabilized before being stained with mouse anti-Myc for Hsp70 (Texas red, Red). Yellow illustrates overlap between Hsp70 (red) and the STCH (green). Bars, 5 μm.

The potential significance of our findings is that the interaction of STCH, a constitutively expressed member of the Hsp70 family, with NKCC2 at the ER and/or cis-Golgi could be very important for the ER quality control and the ERAD of the co-transporter. These findings are of particular interest, because previous reports showed that the cytoplasmic, stress-inducible, and the most studied members of the Hsp70 family Hsp-70-1, commonly called Hsp70, is also involved in the ER quality control of several proteins [33,34,45,46,47,48]. This prompted us to study also the effect of this stress-inducible Hsp70 on NKCC2 biogenesis. Consequently, we first checked whether NKCC2 might also interact with Hsp70. To this end, cells were transiently transfected with Myc-NKCC2 construct either singly or in combination with Hsp70 which is also tagged with Myc. Cell lysates were incubated with anti-Hsp70 antibodies, and the resultant immunoprecipitates were resolved by SDS-PAGE and Western blot using anti-Myc. Interestingly, similar to the data obtained with STCH, these CO-IP experiments revealed Hsp70 interacts also with NKCC2 and that the interaction implicates mainly the immature form of the co-transporter (Figure 6A, *lane 3*).

To check whether Hsp70 interaction with NKCC2 takes place also at the ER, we visualized the subcellular distribution of EGFP-NKCC2 and Myc-Hsp70 by immunofluorescence microscopy. As shown in Figure 6B, EGFP-NKCC2 (green) co-localized with Myc-Hsp70 (red), indicating that these two proteins share overlapping subcellular localization. Interestingly, similar to STCH, Hsp70 protein displayed also co-localization with the ER marker calnexin, which in agreement with the notion that the interaction engages mainly the immature and ER resident form of NKCC2. Interestingly, in cells co-expressing GFP-STCH and Myc-HSP70, these two NKCC2 binding partners appeared also to share some overlapping subcellular localization. Collectively, these data provide evidence, that similar to the non-inducible Hsp70-like protein STCH, the stress-inducible Hsp70 interacts also with the immature form of NKCC2, an interaction that takes place very likely at the cytoplasmic side of the ER.

### 2.7. Differential Regulation of NKCC2 and It Disease Causing Mutants with Exogenous STCH and Hsp70

**Figure 7:**
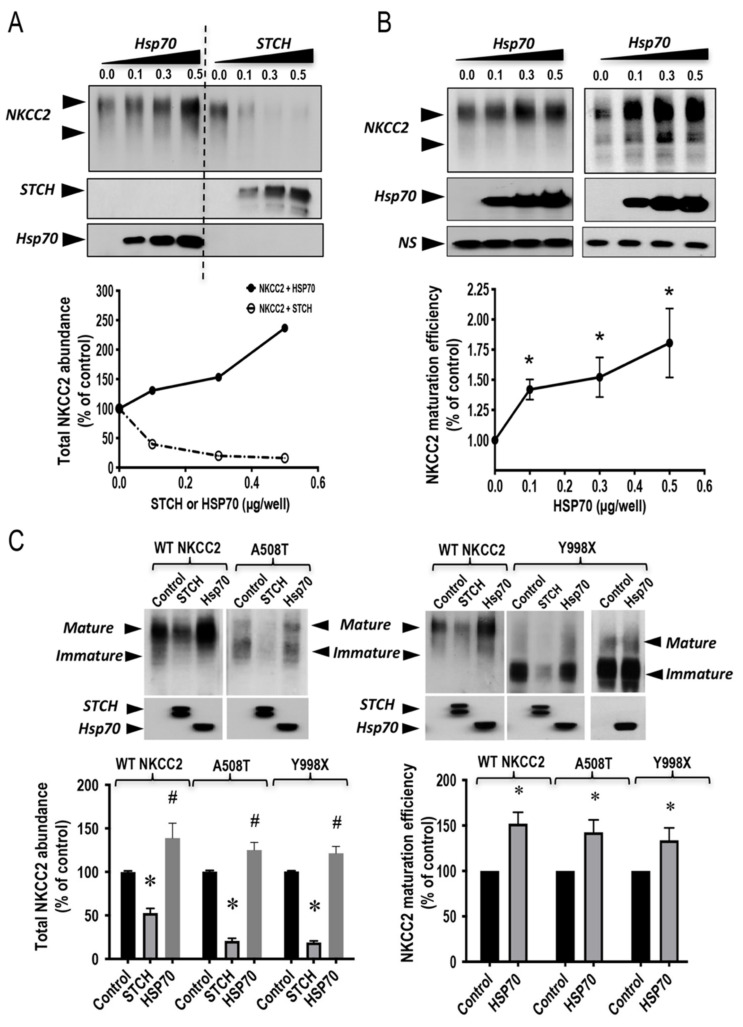
STCH and Hsp70 differentially regulate NKCC2 and its disease-causing mutants. (**A**) Representative immunoblot of two independent experiments showing opposite effects of STCH and Hsp70 on NKCC2 protein abundance. HEK cells were co-transfected with Myc-NKCC2 (0.1 μg/well) and increasing amounts of GFP-STCH (0.1–0.5 μg/well) or Myc-Hsp70 (0.1–0.5 μg/well) as indicated. NKCC2, Hsp70, and STCH proteins were detected by immunoblotting with anti-Myc and anti-GFP anti-bodies. **Lower panel**, densitometric analysis of total NKCC2 proteins. Data are expressed as a percentage of control (*n* = 2). (**B**) Hsp70 co-expression increases NKCC2 maturation efficiency. **Upper panel**, two representative immunoblots illustrating the effect of Hsp70 overexpression on NKCC2 protein abundance and maturation in HEK cells. Cells were transfected with Myc-NKCC2 alone (0.1 μg/well) or in the presence of Myc-Hsp70 (0.1–0.5 μg/well). Then, 24–48 h post-transfection, total cell lysates were subjected to immunoblot analysis for NKCC2 and Hsp70 proteins using anti-Myc. *NS*, a non-specific band illustrating the equal loading of protein extracts. **Lower panel**, densitometric analysis of the maturation efficiency (ratio of the mature vs. immature form of NKCC2) of WT NKCC2 in the presence or absence of Hsp70. Data are expressed as percentage of control ± S.E. Each point represents mean ± SE from three independent experiments (*n* = 3). *, *p* < 0.05 versus control. (**C**) Differential regulation of NKCC2 mutants by STCH and Hsp70. HEK cells were transiently transfected with NKCC2 or BS1 mutants (A508T or Y998X) in the presence or absence of STCH or Hsp70, as indicated. NKCC2, Hsp70, and STCH proteins were detected by immunoblotting with Myc antibody and anti-GFP. **Lower right panel**, densitometric analysis of total NKCC2 proteins. Data are expressed as a percentage of control. * and #, *p* < 0.05 versus control. NKCC2 alone, *n* = 6. NKCC2 with STCH, *n* = 5. NKCC2 with Hsp70, *n* = 5. A508T alone, *n* = 4. A508T with STCH, *n* = 3. A508T with Hsp70, *n* = 3. Y998X alone, *n* = 6. Y998X with STCH, *n* = 3. Y998X with Hsp70, *n* = 4. **Lower left panel**, densitometric analysis of the maturation efficiency (ratio of the mature vs. immature form of NKCC2) of WT NKCC2 (*n* = 3), A508T (*n* = 3), and Y998X (*n* = 4), the presence or absence of Hsp70. Data are expressed as percentage of control ± S.E. *, *p* < 0.05 versus control.

The interaction of the stress-inducible Hsp70 with the immature form of NKCC2 opens the possibility for its involvement also in the regulation of NKCC2 biogenesis. Consequently, we next compared the effect of STCH with that of Hsp70 on the expression of NKCC2 protein. To this end, HEK cells were transfected singly with NKCC2, or in combination with STCH or Hsp70. As shown in Figure 7A, when increasing amounts of STCH or Hsp70 (0.1–0.5 μg/well) were co-expressed with NKCC2 (0.1 μg/well), NKCC2 expression was, again, heavily reduced by STCH, whereas it was markedly increased by Hsp70, in a dose-dependent manner. Interestingly, these data revealed also, that Hsp70 co-expression effect on the mature form of NKCC2 is greater than that observed on the immature form of the co-transporter. Consequently, as can be seen in Figure 7B, Hsp70 co-expression increased the ratio of the mature vs. immature form of NKCC2, in a dose-dependent fashion strongly indicating that Hsp70 increases the maturation efficiency of the co-transporter. Hence, these data further support, per se, the notion that STCH induced-downregulation of NKCC2 is specific. Most importantly, they provide evidence that the non-inducible Hsp70-like protein STCH and the stress-inducible Hsp70 differentially regulate NKCC2 biogenesis.

To further corroborate the notion that STCH and Hsp70 differentially regulate the co-transporter biogenesis, we next compared the effects of STCH and Hsp70 on the expression of A508T and Y998X, two previously reported Bartter syndrome type 1 mutations [29,49,50]. Importantly, when compared to its action on WT NKCC2, STCH co-expression had a more profound effect on A508T and Y998X mutants suggesting that STCH is involved in the ERAD of NKCC2 disease causing mutants. As shown in Figure 7C, STCH and Hsp70 had opposite effects on WT NKCC2 but also on A508T and Y998X. Most importantly, similar to WT NKCC2, Hsp70 co-expression seemed to improve also the maturation efficiency of A508T and Y998X as judged by the ratio of the mature form vs. the immature form of the co-transporter (Figure 7C, **lower right panel**), which is per se, a clear and additional indication that STCH and Hsp70 have differential effects on the co-transporter expression and maturation.

### 2.8. Endogenous Stress-Inducible HSP70 and STCH Differentially Regulate NKCC2 Expression

**Figure 8:**
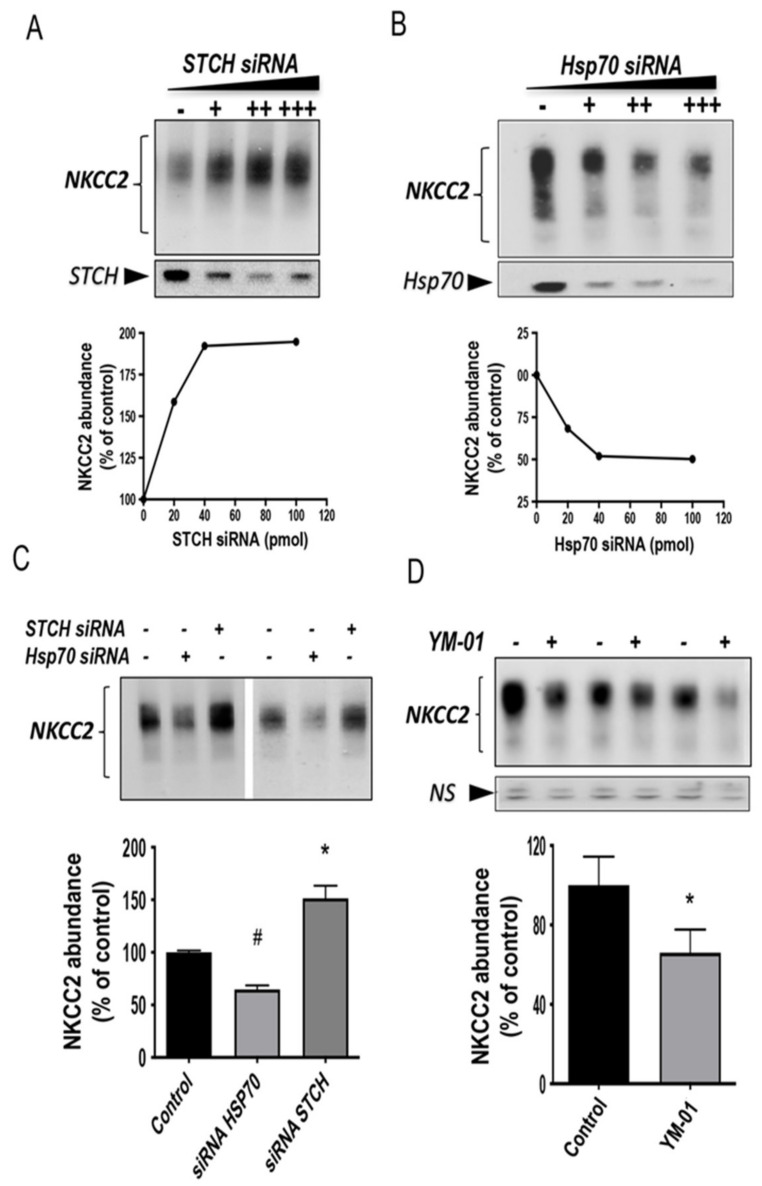
Differential regulation of NKCC2 expression by endogenous Hsp70 and STCH. (**A**,**B**) Knockdown of endogenous STCH or Hsp70 in HEK cells regulate total NKCC2 protein abundance in a dose-dependent fashion. **Upper panels**, representative immunoblot analysis of two independent experiments illustrating the effect of STCH knockdown (**upper left panel**) or Hsp70 knockdown (**lower right panel**) on NKCC2. HEK cells were transfected with NKCC2 in the absence (-) or presence of an increasing amount (+, ++, +++) of specific STCH siRNA or Hsp70 siRNA. 48 h post-transfection, total cell extract from each sample was run on a parallel SDS gel and Western blotted for total NKCC2 expression. NKCC2 proteins were detected by immunoblotting with Myc antibody. **Lower panels**, densitometric analysis of total NKCC2 proteins. Data are expressed as percentage of control (*n* = 2). (**C**) Opposite effects of STCH and Hsp70 knockdowns on total NKCC2 protein. Representative immunoblot analysis showing the effect of Hsp70 knockdown and STCH knockdown on total NKCC2. HEK cells were transfected with NKCC2 in the absence (−) or presence of specific Hsp70 siRNA (+) or STCH siRNA (+). Then, 48 h post-transfection, total cell extract from each sample was subjected to immunoblotting analysis. **Lower panel**, densitometric analysis of total NKCC2 proteins. Data are expressed as percentage of control. Each point represents mean ± SE from four independent experiments (*n* = 4). #, *p* < 0.05 versus control. *, *p* < 0.003 versus control. (**D**) Effect of Hsp70 inhibitor YM-01 on NKCC2. HEK cells transfected with NKCC2 were treated with 1 μM of YM-01 (+) or without (−) overnight before cell lysis and immunoblotting using anti-Myc for NKCC2. *NS*, a non-specific band illustrating the equal loading of protein extracts. **Lower panel**, densitometric analysis of total NKCC2 proteins. Data are expressed as percentage of control. Each point represents mean ± SE from three independent experiments (*n* = 3). *, *p* < 0.05 versus control.

The differential effects of Hsp70 and STCH on NKCC2 biogenesis strongly suggest, per se, that their action on the co-transporter are specific. To further corroborate the differential regulation of NKCC2 by STCH and Hsp70 and the specificity of their effects on the co-transporter, we next examined the role of endogenous STCH and Hsp70 on NKCC2 protein expression. Consequently, we studied the effect of STCH and Hsp70 knockdown on NKCC2 using small interference RNA (siRNA). To this end, a sequential transfection in HEK cells was performed. Cells were first transfected with siRNAs for at least 24 h, before transfection with Myc-NKCC2 plasmids for 48 h. As illustrated in Figure 8, upon STCH knockdown, the steady state level of NKCC2 was increased in a dose dependent fashion (Figure 8A). In contrast, Hsp70 knockdown had the opposite effect by decreasing NKCC2 protein abundance in a dose-dependent manner (Figure 8B), illustrating again the specificity of STCH and Hsp70 actions on the co-transporter. Given that a plateau was reached with the dose of 40 pmol of each siRNA (Figure 8A,B), this dose was used to study in parallel the effects of STCH and Hsp70 knock-down on total NKCC2 protein abundance. As can be seen in Figure 8C, when the dose of 40 pmol of each siRNA was used, STCH know-down increased NKCC2 protein abundance by 52% (*p* < 0.05), whereas HSP70 know-down decreased it by 35% (*p* < 0.003), illustrating again the reciprocal control of NKCC2 by STCH and Hsp70. To further support the notion that endogenous Hsp70 promotes NKCC2 biogenesis, we also tested the effect of YM-01, an allosteric inhibitor of Hsp70 [51,52]. As illustrated in Figure 8D, YM-01 reproduced the effect of Hsp70 knock-down by downregulating the steady state level of NKCC2 (−34 %, *p* < 0.05). Hence, these data are fully in agreement with a role of endogenous of Hsp70 and STCH in the regulation of NKCC2 protein expression. Most importantly, these results are consistent with STCH and Hsp70 having differential and antagonistic effects with regard to NKCC2 biogenesis.

## 3. Discussion

This study was conducted to gain insight into the molecular mechanisms underlying the regulation of NKCC2 transit through the ER. Here, we have identified STCH and Hsp70 as two novel interacting partners of the immature and ER-resident form of NKCC2. In contrast to Hsp70, the implication of STCH in the ERAD of misfolded proteins has never been directly investigated. Here, we have found that STCH and Hsp70 differentially regulate the protein expression of NKCC2 and its diseasing causing mutants. Indeed, whereas STCH enhances the ERAD of NKCC2, Hsp70 exerts the opposite effect by promoting the maturation of the co-transporter. Interestingly, the ERAD of NKCC2 mediated by STCH involves both the proteasome and the lysosome pathways. The reciprocal control of NKCC2 stability and maturation by STCH and Hsp70 may open new avenues in the treatment of diseases related to abnormal NKCC2 trafficking.

Most members of the Hsp70 family belong to the Dnak family and they are recognized to play protective effects through several mechanisms, such as assisting for proper folding of misfolded proteins and degradation of damaged proteins [33,34,53]. The stress 70 protein chaperone (STCH), a member of the Hsp70 family, is constitutively expressed in all human cell types and shares a high degree of amino acid identity with Hsp70 [32]. Unlike other members of the DnaK subfamily, STCH contains a unique hydrophobic signal sequence and lacks a carboxyl-terminal peptide-binding domain [32]. Many studies have associated the *STCH* gene to several brain diseases such as Alzheimer disease, epilepsy, autism but also to gastric cancer [35,36,37,38]. Despite these insights in STCH biological functions, the data relying on its role at the molecular level remain scarce. Kaye et al. has identified an interaction between STCH and the ubiquitin-like protein UBQLN2, termed CHAP1/DSK2, which is known to mediate the proteasomal targeting of misfolded proteins for degradation by binding to their polyubiquitin chains and by interacting (via ubiquitin-like domain) with the subunits of the proteasome [54]. UBQLN2 plays also a role in the ERAD pathway, providing a link between polyubiquitinated ERAD substrates and the proteasome. Although the precise molecular mechanisms remain unclear at this stage, the identification of ubiquitin-linked proteins that bind to a conserved peptide motif within the ‘core ATPase’ STCH molecule suggests a broader role for this Hsp70 like protein in regulating specialized cellular events. However, whether STCH has a direct role in ERAD of misfolded proteins has never been directly addressed. In the present study, we showed evidence for specific binding of STCH with NKCC2. Most importantly, we showed for the first time, that this interaction is required for efficient degradation of NKCC2 by the ERAD pathway.

We previously demonstrated that ERAD and export from the ER constitute the limiting step in the maturation and cell surface expression of NKCC2 [27,28,29,30]. In the present work, the function of STCH in the ERAD of NKCC2 was first suggested by co-immunoprecipitation studies that demonstrated that the interaction involves mainly the immature, ER-resident form of NKCC2. Accordingly, immunocytochemistry studies showed that STCH mainly co-localizes with NKCC2 at the ER. To further support the role of STCH in the ERAD of NKCC2, we have shown that the overexpression of STCH or its inhibition using siRNA approach, was associated with an increase or a decrease in the expression and the degradation of the immature form of the co-transporter, respectively. It is important to note that Bae et al. [41] have identified a positive role of STCH in the modulation of membrane trafficking of two acid/base transporters NBCe1-B and NHE1, by increasing their cell surface expression and activity. It is well known that a single chaperone might be involved in either folding or degrading a given substrate that travels through the ER [26]; STCH is no exception and its effect is substrate-dependent. Hence, it may have a protective role for NBCe1-B and NHE1 but a destructive one for NKCC2 during ERAD. Moreover, we have shown that STCH effects on the immature form of NKCC2 were predominantly prevented in the presence of the proteasome inhibitor MG132, indicating that STCH targets most of the immature form of the co-transporter to the proteasome-dependent ERAD pathway. Interestingly, full recovery of NKCC2 expression was achieved only by using MG132 combined with chloroquine, an inhibitor of lysosomal function. Thus, we show here for the first time that the lysosome machinery is also implicated in the proteolysis of NKCC2 by playing a complementary role with the proteasome in the degradation of misfolded NKCC2. The role of additional ER quality control mechanisms, defined as ER-to-lysosome-associated degradation (ERLAD) pathways, is progressively emerging, as some misfolded proteins evade the ERAD factors for quality control or they can form aggregates that cannot be dislocated across the membrane [55,56]. These misfolded ER clients, which are not suitable for ERAD must be cleared from the ER through the lysosome alternative by autophagic or non-autophagic pathways. It has been shown that Hsp70 chaperones have a dual function in protein degradation, as they can select misfolded proteins for either the general protein degradation pathways, the proteasome system, or the lysosome degradation machinery [33,34,45,46,47,48,57,58]. Our present data demonstrate that immature NKCC2 is recognized by STCH, a component of the Hsp70 system, and degraded by two different pathways that function in a complementary way to regulate NKCC2 turnover. We believe that our characterization for the first time of the ERLAD as another degradation pathway of NKCC2, as well as ERAD, supports and offers a new mechanistic explanation of the ER quality control of NKCC2. How this is regulated awaits further clarification.

ERAD machinery has been reported to function primarily in the ER where ER α-1, 2-mannosidase I (ERManI) is capable of initiating the ERAD by promoting the first mannose trimming step of numerous misfolded N-glycosylated protein [59,60,61]. However, proteins that exhibit a severe misfolded conformation can traffic to post-ER compartments [62,63]. These findings suggest that the ER’s ability to retain misfolded proteins is limited and therefore requires a post-ER control system to prevent premature secretion of escaped misfolded immature proteins. Evidence obtained from several studies support the involvement of the Golgi complex as a component of ERAD. Indeed, it has been demonstrated that a number of ERAD substrates undergo vesicular cycling through early Golgi compartments, and that the three-traditional mammalian Golgi-localized α-1, 2-mannosidases (IA, IB, and IC) contribute to the intracellular degradation of misfolded glycoproteins [63,64,65,66]. Furthermore, ERManI, which was predicted to function in the ER, was also localized to the Golgi complex in mammalian cells, where it contributes to a Golgi-based quality control checkpoint that facilitates the retrieval of captured ERAD substrates back to the ER [65,67]. Interestingly, we have shown in our current study, that upon treatment with mannosidase I inhibitor kifunensine, the effects of STCH on NKCC2 expression were abolished. Given the fact that kifunensine inhibits trimming of mannose residues in the endoplasmic reticulum (ER) and in the Golgi compartment, these results suggest that the STCH-enhanced degradation of misfolded NKCC2 requires mannose trimming by α-mannosidase I to target ER misfolded NKCC2 as well as escaped misfolded NKCC2 in the Golgi for ERAD. STCH is expected to be mainly located in the ER, a prediction confirmed also by our experiments. However, in contrast to ER resident proteins, STCH does not encode a consensus ER retention signal such as the tetrapeptide KDEL [32] opening therefore the possibility that STCH may exit the ER. In support of this notion, Otterson et al. showed that STCH is localized to the cellular cytoplasm in a pattern that resembled, but was not identical to BiP, an ER resident protein [32]. Accordingly, we show here that not all STCH related immunofluorescence signal was found in the ER. In fact, the remaining staining was dispersed in a perinuclear Golgi like region. Most importantly, we found colocalization of STCH with GM130, strongly suggesting that in addition to its detection at the ER, this Hsp70-like protein is also present the cis-Golgi network. These findings are of great interest because they are consistent with the expression of STCH as a Man-6 phosphorylated glycoprotein [42] and its sensitivity to endo-H digestion (Figure 2B). Indeed, phosphorylation of mannose residues within the 1,6 branch prevents high mannose-type units from being converted to Endo H resistant structures by preventing mannosidase action [43,44]. However, despite being Man-6 P phosphorylated [42], we did not detect any colocalization of STCH with the lysosome marker LAMP2, which clearly indicates that although some STCH proteins exit the ER and then become Man-6-phosphorylated in cis-Golgi apparatus, they were retained in the cis-Golgi network and/or retrieved back to the ER prior to encountering the MPRs in the trans-Golgi. In sum, these observations support a model in which STCH role in the ERAD of immature NKCC2 requires mainly its residence in the ER. Moreover, they open the possibility that STCH contributes not only in the retention and degradation of misfolded proteins at the ER, but also in the retention, the recycling, and ERAD of misfolded proteins that initially escape protein quality control surveillance within the ER.

Among the members of 70 kDa heat shock proteins, Hsc70 (heat shock cognate protein, Hsp73/HSPA8) and Hsp70 (Hsp72/HSPA1A) have been extensively studied and have distinct biological functions despite their high sequence homology [33,34,53]. Consequently, the roles of Hsp70 and Hsc70 in the ERAD of several transmembrane proteins such CFTR, NCC, ENaC, and HERG have been thoroughly investigated [24,45,46,47,48,57]. Under cellular stress conditions, it is assumed in general that Hsc70 associates with newly synthesized misfolded proteins to induce their ER associated degradation by the proteasome. In contrast, Hsp70 seems to preferentially promote the biogenesis of newly synthesized proteins by facilitating their protein refolding after cellular stress [68]. In agreement with these notions, several reports demonstrated that Hsp70 and Hsc70 exert opposite effects on the stability and maturation of hERG and ENaC proteins through their association with their immature forms at the ER [45,48]. This is of great interest because similar to STCH, Hsc70 is also constitutively expressed [69]. Hence, we hypothesized that STCH and the stress-inducible Hsp70 may also have differential effects on the stability and maturation of NKCC2. Importantly, we found indeed that similar to Hsc70, the effect of STCH was opposite to that of Hsp70 not only on WT NKCC2 but also on its disease-causing mutants i.e., STCH promotes NKCC2 degradation, whereas Hsp70 promotes the co-transporter maturation. Similar to STCH, the main site of action of Hsp70 appeared to be mainly the ER given that both chaperones associate only with the immature form of the co-transporter. Interestingly, our immunocytochemistry experiments revealed overlap of Hsp70 and STCH, which is consistent with the idea that these two chaperones exert opposite and antagonist effect on the same substrate, which in our case, is NKCC2. Obviously, further studies are required to determine the mechanisms underlying these differential effects and whether these effects are also observed native TAL cells. Of note, while under ER stress conditions, Hsp70 preferentially promotes, in general, the stabilization and proper protein folding of newly synthetized proteins, it promotes actually their degradation under non-stress conditions [26,68]. This is of particular interest because our experimental setting i.e., heterologous overexpression of secretory and membrane proteins (NKCC2) causes ER stress which is therefore consistent with the protective role of Hsp70 during the ERAD of the co-transporter under these conditions [70,71,72]. With regard to the mechanisms underlying the effect of Hsp70 on protein maturation, it is worth mentioning that Chanoux et al. demonstrated that Hsp70 overexpression increases the association of ENaC with the Sec24D cargo recognition component of coat complex II, which carries protein cargo from the endoplasmic reticulum to the Golgi [73]. It is therefore tempting to speculate that Hsp70 overexpression stabilizes NKCC2 at the ER and promotes also its maturation and trafficking by increasing its association with Coat Complex II and its exit from Endoplasmic Reticulum. Undeniably, Hsp70 cannot work in isolation to promote NKCC2 biogenesis. Appropriately, one may reasonably postulate that Hsp70 works in concert, sequentially or simultaneously, with other NKCC2-binding proteins and ERAD components such as MAGE-D2 and Hsp40 to regulate the ER quality control and its associated protein degradation of the co-transporter. Indeed, it is well known that the function of Hsp70 in the ERAD pathway involves Hsp40 [33,57,58]. Most importantly, we previously provided evidence that MAGE-D2 protects the co-transporter against ER associated degradation and promotes its maturation and cell surface expression, an effect that is very likely to be mediated by Hsp40 [27]. Likewise, given that we previously showed the presence of OS9 mediated ER associated degradation of NKCC2 [28], one may also postulate that STCH and OS9 work in concert to exert the opposite effect by promoting efficient degradation of the co-transporter and its folding mutants. Obviously, further experiments are needed to uncover the precise mechanisms behind these processes.

In summary, we identified Hsp70-like STCH and the stress inducible Hsp70 proteins as two novel bindings partners of the immature and ER-resident form of NKCC2. Our results provide evidence that STCH and Hsp70 exert opposite effects on the stability and maturation of the co-transporter. In addition, they show that besides the proteasome-dependent ERAD, the ER quality control of NKCC2 mediated by STCH, involves also the ER-to-lysosome-associated degradation pathway, revealing therefore a new regulatory mechanism governing the co-transporter biogenesis. To the best of our knowledge, this is also the first report demonstrating that STCH is directly involved in the ERAD of misfolded proteins. Given the dual role of chaperones such as the members of the Hsp70 heat shock family in ERAD pathways [26], an attractive idea is that biochemical and/or molecular manipulation of their cellular levels might allow proper folding, and consequently partial rescue of function of mutant proteins. Accordingly, enhancing the ability of chaperones, such Hsp70 and Hsp40 to stabilize NKCC2 and assist its proper folding might at least partially rescue trafficking of its folding mutants, and consequently restore their function at the plasma membrane. Furthermore, reducing the ability of chaperones, such as STCH and OS9 to promote the ERAD NKCC2, might increase the cellular levels of mutant proteins available to be rescued. Ultimately, the comprehensive characterization and identification of the molecular specific determinants of ERAD NKCC2 might help to provide a foundation for the development of therapeutic strategies targeting co-transporter trafficking from the ER to the plasma membrane.

## 4. Materials and Methods

### 4.1. Materials

All chemicals were obtained from Sigma unless otherwise noted. Penicillin and streptomycin were from Invitrogen. Subclonings were carried out with the following vectors: 1) pGKT7 (Clontech, Saint-Germain-En-Laye, France), 2) pCMV-Myc (Clontech), 3) pEGFP-C2 (Clontech), 4) pcDNA3.1/V5-His-TOPO (Invitrogen, Paris, France).

### 4.2. Yeast Two-Hybrid Assay

Yeast two-hybrid (Y2H) screening was performed as described previously in details [39]. Briefly, the cDNA fragment encoding the distal region of the NKCC2 C terminus (last 195 aa) was cloned in-frame with the GAL4 DNA-binding domain in pGBKT7-BD and transformed into the yeast strain AH109. AH109 expressing the bait was then mated with the Y187 yeast strain pretransformed with a human kidney cDNA library constructed in the pACT2-AD vector. Mated yeast cells were first grown on low stringency selection plates (−Leu, −Trp, −His) and then on high stringency selection plates (−Leu, −Trp, −His, −Ade). Colonies were tested for β-galactosidase activity, and DNA from positive clones encoding the putative interacting proteins was isolated from yeast cells using the RPM yeast plasmid isolation kit (BIO 101 Systems, Illkirch, France). Prey plasmids were rescued by transformation into DH5 α bacteria (Invitrogen) and isolated using a Qiagen kit. cDNA plasmids were then sequenced and assessed using the BLAST program.

### 4.3. Plasmid Constructions and Site Directed Mutagenesis

The cDNA encoding mouse NKCC2 was fused at the C-terminal end of Myc (Myc- NKCC2) or EGFP (EGFP-NKCC2) using pCMV-Myc and pEGFP-C2 vectors, respectively [39]. The pcDNA3 plasmid encoding Myc-Hsp70 [46] was a kind gift from Jeffrey L Brodsky and Arohan R Subramanya (University of Pittsburgh, PA, USA). The pEGFP-C1 plasmid encoding GFP-STCH [41] was a kind gift from Kyungpyo Park (Seoul National University and Dental Research Institute, Seoul, Korea).

### 4.4. Cell Culture

Opossum kidney cells (OKP cells) were grown in DMEM complemented with 10% fetal bovine serum (Invitrogen), penicillin (100 U/mL), and streptomycin (100 U/mL) at 37 °C in a humidified atmosphere containing 5% CO_2_. Human embryonic kidney (HEK) 293 cells were maintained in DMEM media supplemented with 10% fetal bovine serum and 1% penicillin/streptomycin. For DNA transfection, cells were grown to 60–80% confluence on plastic culture dishes and then were transiently transfected for 5 h with plasmids using Lipofectamine plus kit according to manufacturer’s instructions (Invitrogen). For protein degradation assays, transiently transfected cells were treated with MG132 (2 μM) or chloroquin (100 μM) for 6 h prior to cell lysis.

### 4.5. Protein Preparation, Immunoblotting, and Immunoprecipitation

16–48 h post-transfection, cells were washed with cold PBS and lysed in 0.2 or 0.5 mL lysis buffer (120 mM Tris/Hepes, pH 7,4; 150 mM NaCl, 5 mM EDTA, 3 mM KCl; 1% (*v*/*v*) Triton X-100) containing protease inhibitors (Complete Roche 1697498, Meylan, France). Samples were harvested and centrifuged at 16,000 rpm for 15 min at 4 °C. Protein expression levels were assessed after normalizing and loading equal amounts of total protein for 7.5% SDS-PAGE separation and immunoblotting with the antibodies of interest. For immunoprecipitation, cells were solubilized with lysis buffer containing 0.4 M NaCl; 0.5 mM EGTA; 1.5 mM MgCl_2_; 10 mM Hepes, pH 7.9; 5% (*v*/*v*) glycerol; 0.5% (*v*/*v*) Nonidet P-40) and protease inhibitors (Complete, Roche Diagnostics, Meylan France). Immunoprecipitation was carried out using the primary antibody of interest, and affinity purification using protein G-agarose beads (Dynal). After incubation with protein G-agarose beads for 1 h at room temperature, the immunocomplex was washed three times in PBS (Invitrogen). The protein samples were boiled in loading buffer, run on gradient 7.5% SDS-polyacrylamide gels, probed with primary antibodies of interest and horseradish peroxidase-conjugated secondary antibody, according to standard procedures. Proteins were visualized by enhanced chemiluminescence detection (PerkinElmer Life Sciences, Villebon-sur-Yvette, France) according to the manufacturer’s instructions.

### 4.6. Immunocytochemistry

24–48 h post-transfection, confluent cells were washed twice with PBS^++^ (pH 8, 1 mM MgCl_2_, and 0.1 mM CaCl_2_). Cells were then fixed with 2% paraformaldehyde in PBS for 20 min at room temperature, incubated with 50 mM NH_4_Cl, permeabilized with 0.1% Triton X-100 for 1 min and incubated with DAKO (antibody diluent with background-reducing components) for 30 min to block no specific antibody binding. Fixed cells were incubated for 1 h at room temperature with the primary antibodies at appropriate dilution in DAKO. Mouse anti-Myc was visualized with Texas Red-coupled or Alexa Fluor 647 conjugated secondary antibodies (Thermo Fisher Scientific, Paris, France). Rabbit anti-calnexin (Abcam, Paris, France) was visualized with FITC-coupled antibodies (Themo Fisher Scientifc). Anti-Giantin (Abcam), anti-GM130 (Abcam), and LAMP2 (Abcam) were with Alexa Fluor 555 conjugated secondary antibodies (Thermo Fisher Scientific). Cells were then washed with PBS and mounted with Vectashield.

### 4.7. siRNA Knockdown

The siRNAs for STCH (HSPA13) and Hsp70 (HSPA1A) siRNAs were purchased from Dharmacon as ON-TARGET plus SMART pools (L-015679-00-0005 and L-005168-00-0005, respectively). HEK cells were first transfected with control or specific siRNA with Lipofectamine RNAiMAX (Invitrogen, Paris, France) using the manufacturer’s specifications. One day after siRNA transfection, cells were transfected with NKCC2 plasmids. Then, 48 h after NKCC2 transfection, cell lysates were analyzed for each protein using the indicated antibodies.

### 4.8. Statistical Analyses

Results are expressed as mean ± SE. Differences between means were evaluated using paired or unpaired *t* test or ANOVA as appropriate. *p* < 0.05 was considered statistically significant.

## Data Availability

All data are contained within the manuscript.
